# GPR37 modulates body weight and insulin sensitivity in a sex-biased manner

**DOI:** 10.1371/journal.pone.0349406

**Published:** 2026-05-28

**Authors:** Mariam Ahmed, Mariela Nunez Santos, Karen Abdelsayed, Corrine Liu, Nimco Xuseen, Pokuaa Adwoa Boakye, Sharon Owino

**Affiliations:** 1 Neuroscience Program, Smith College, Northampton, Massachusetts, United States of America; 2 Department of Pharmacology and Toxicology, Morehouse School of Medicine, Atlanta, Georgia, United States of America; National Institute of Child Health and Human Development (NICHD), NIH, UNITED STATES OF AMERICA

## Abstract

Metabolic disorders are a growing public health concern in the United States, with approximately 40% of the population living with obesity. The urgent need for novel therapeutic targets has driven interest in G protein–coupled receptors (GPCRs), a diverse group of seven-transmembrane receptors that regulate various physiological processes and represent a significant portion of current drug targets. In this study, we investigated the role of GPR37, a brain-enriched orphan GPCR, in systemic glucose regulation. Using heterozygous *Gpr37 + /-* mice, we assessed body weight, glucose tolerance, and insulin sensitivity. Male *Gpr37 + /-* mice exhibited significantly reduced body weight, an enhanced metabolic response to fasting, and increased insulin sensitivity compared to wild-type controls. These findings indicate that reduced *Gpr37* gene dosage is associated with metabolic efficiency, particularly in the regulation of glucose metabolism, and reveal a previously unrecognized sex-biased role for GPR37 in systemic energy homeostasis. Taken together, these data suggest that GPR37 contributes to metabolic regulation and represents a candidate pathway for further pharmacological and tissue-specific study.

## Introduction

Metabolic disorders involving glucose metabolism, such as obesity and diabetes, are rising at an alarming rate in the United States, posing significant public health challenges. A recent study found that only 12% of American adults are metabolically healthy, even among individuals of normal weight [1]. Currently, approximately 40% of Americans live with obesity [2], while globally, an estimated 589 million adults aged 20–79 are affected by diabetes [3]. The increasing incidence and reduced quality of life associated with these disorders underscore the urgent need for new therapeutic targets.

G protein–coupled receptors (GPCRs), which account for over thirty percent of approved drug targets, are increasingly recognized as promising therapeutic candidates for metabolic diseases, due to their critical roles in regulating fat storage, glucose homeostasis, insulin sensitivity, nutrient metabolism, and overall energy balance [4–6]. GPR37, a brain-enriched GPCR, primarily expressed in oligodendrocytes, is also detected in ghrelin-producing cells of the gut and in the liver, suggesting a potential role in regulating systemic metabolic regulation [7]. Unlike its homolog GPR37L1, whose metabolic role appears minor [8], GPR37’s role in glucose metabolism and insulin sensitivity remains unknown.

To examine how glucose metabolism is affected in GPR37 heterozygous mice (*Gpr37 + /-*), we compared metabolic outcomes between wild-type (WT), and *Gpr37 + /-* mice. Glucose and insulin tolerance tests were performed to evaluate metabolic responses in both male and female mice. *Gpr37 + /-* mice exhibited increased insulin sensitivity, reduced body weight, and a more pronounced fasting-induced decrease in blood glucose levels. Notably, these effects were more evident in male mice, suggesting a potential sex-biased contribution of GPR37 to metabolic homeostasis.

## Materials and methods

All animal procedures were conducted in accordance with the guidelines established by the Smith College Institutional Animal Care and Use Committee (IACUC). The protocol was reviewed and approved by Smith College IACUC (Protocol Number: ASAF#22R-SO-301). All animal procedures were approved by the Institutional Animal Care and Use Committee and conducted in accordance with NIH guidelines. Animals were handled by trained personnel, and all procedures were performed to minimize discomfort and stress; mice were euthanized by CO_2_ inhalation followed by cervical dislocation as a secondary method consistent with AVMA guidelines, and no surgical procedures requiring anesthesia or analgesia were performed in this study. GPR37 knock-out mice (Gpr37 *-/-*) were generously donated by Dr. Randy Hall (Emory University) and backcrossed with wild-type mice (Jackson Laboratory) to generate WT and Gpr37 + /- mice. Six-month-old male and female Gpr37 + /- mice and their WT littermates were used for all metabolic assessments. Mice were group-housed in a temperature- and humidity-controlled facility under a 10-hour light/14-hour dark cycle, with ad libitum access to standard chow and water. Following a 6-hour fast, glucose and insulin tolerance were evaluated using an intraperitoneal (i.p.) glucose tolerance test (GTT) or an i.p. insulin tolerance test (ITT). During the GTT, mice were administered a bolus of 20% D-glucose solution (1.5 g/kg body weight) following which a calibrated glucometer was used to measure blood glucose levels via the tail vein at 0, 30, 60, 90, and 120 minutes post-injection. During the ITT, mice received an i.p. injection of insulin (0.5 U/kg body weight), and blood glucose levels were measured at 0, 15, 30, 60, and 120 minutes post-injection. All data were analyzed using two-way ANOVA or unpaired two-tailed t-tests where appropriate, with statistical significance defined as p < 0.05. Two-way ANOVA models included Sex, Genotype, and Sex × Genotype interaction terms where applicable. Sample sizes are reported in the figure legends, and data are presented as mean ± SEM.

## Results

### Reduced body weight and enhanced fasting glucose response in male *Gpr37 + /-* mice

**Fig 1:**
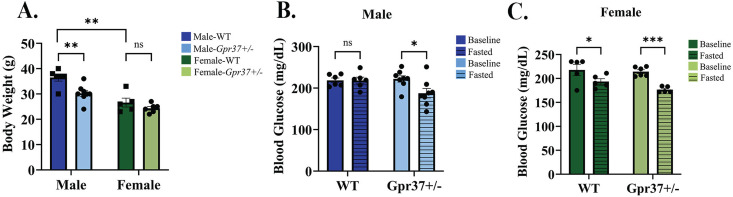
Reduced body weight and enhanced fasting glucose response in *Gpr37 + /-* mice. A: Baseline weights of 6-month-old male and female mice. B: Baseline and fasted blood glucose levels of male mice. C: Baseline and fasted blood glucose levels of female mice. Data represent n = 5-8 mice per group ± SEM. Statistical significance: *p < 0.05, **p < 0.01, ***p < 0.001., two-way ANOVA or t-test.

To investigate genotype- and sex-dependent effects of GPR37 on body weight, we compared body mass between WT and *Gpr37 + /-* mice. Consistent with known sex differences, female mice exhibited lower body weights than males across genotypes. Interestingly, male *Gpr37 + /-* mice demonstrated a significant reduction in body weight relative to WT males. This genotype-associated difference was not observed in females, indicating a sexually dimorphic effect of *Gpr37* haploinsufficiency on body weight regulation, Fig 1A.

Glucose is a key regulator of energy metabolism, and its levels are tightly controlled through the actions of insulin and glucagon. Dysregulation of glucose homeostasis can lead to metabolic disorders. To evaluate the metabolic state of WT and *Gpr37 + /-* mice, we measured blood glucose levels before and after a 6-hour fast. Our results show that both male (Fig 1B) and female (Fig 1C) *Gpr37 + /-* mice exhibited a significant reduction in blood glucose levels following a 6-hour fast. In contrast, WT male mice did not exhibit  a comparable decrease in blood glucose under the same fasting conditions (Fig 1B). This is in line with previous studies demonstrating that male C57BL6 mice only begin to lower blood glucose levels after 12 hours of fasting [9]. Taken together, these findings suggest that *Gpr37 + /-* mice exhibit an accelerated metabolic response to fasting, particularly in males, and improved glucose regulation.

### Increased insulin sensitivity in male *Gpr37 + /-* mice

**Fig 2:**
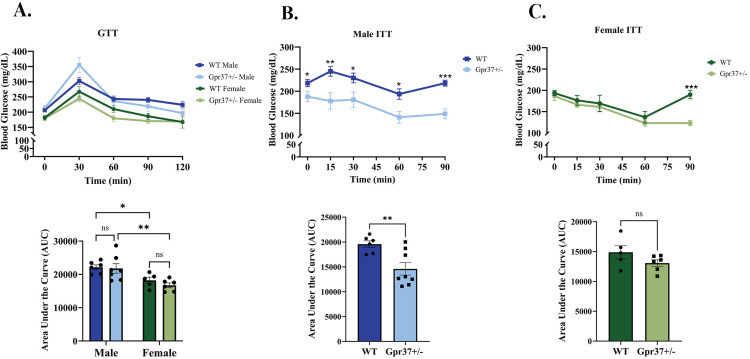
Increased insulin sensitivity in male *Gpr37 + /-* mice. Mice were fasted 6 hours prior to receiving either an i.p injection of glucose (1.5g/kg) or insulin (0.5U/kg) at t = 0 min. A: GTT in male and female WT and *Gpr37 + /-* mice. B: ITT in in male mice. C: ITT in female mice. AUC was calculated for each group. Data represent n = 5-8 mice per group ± SEM. Statistical significance: *p < 0.05, **p < 0.01, ***p < 0.001., two-way ANOVA or t-test.

The glucose tolerance test (GTT) is a metabolic assay used to evaluate glucose clearance and overall glucose homeostasis following a glucose challenge [10]. As expected, female mice displayed enhanced glucose tolerance compared to male mice following glucose administration. However, no significant differences in glucose tolerance were observed between WT and *Gpr37 + /-* mice within either sex (Fig 2A). These findings were supported by area under the curve (AUC) analysis, which confirmed no significant genotype-dependent differences.

The ITT is commonly used to assess insulin sensitivity to insulin by measuring how effectively glucose is cleared from the bloodstream following insulin administration. Male *Gpr37 + /-* mice exhibited a significant and sustained reduction in blood glucose levels compared to WT controls during the ITT, consistent with enhanced insulin sensitivity (Fig 2B). This effect was supported by AUC analysis, which demonstrated a significant reduction in cumulative blood glucose levels in Gpr37 + /- males. This genotype-dependent effect was sex-biased, as no significant AUC differences were observed between WT and *Gpr37 + /-* female mice (Fig 2C). Despite this, both male and female *Gpr37 + /-* mice exhibited delayed glucose recovery, as indicated by a slower return to baseline glucose levels by the 90-minute time point, whereas WT mice fully returned to baseline (Fig 2B, 2C).

## Discussion

GPCRs are critical regulators of metabolic homeostasis. Although previous studies on GPR37L1 have suggested a limited role in glucose regulation [8], the specific metabolic functions of GPR37 remain unknown. In this study, we characterized the metabolic consequences associated with *Gpr37 + /-* mice. Our results demonstrate that *Gpr37 + /-* mice exhibit significantly lower fasting blood glucose levels, indicating an enhanced physiological response to fasting. In male C57BL/6 mice, blood glucose levels usually remain stable during shorter fasting intervals; therefore, the marked reduction observed in *Gpr37 + /-* mice after 6 hours suggests an altered early fasting metabolic response. Notably, male *Gpr37 + /-* mice also displayed reduced body weight and enhanced insulin sensitivity. Taken together, these findings indicate that *Gpr37 + /-* mice exhibit altered systemic metabolic responses, with associated improvements in insulin sensitivity and glucose clearance. The absence of a genotype effect in the glucose tolerance test, despite improved insulin tolerance, likely reflects that these assays measure distinct aspects of metabolic regulation: GTT assesses systemic glucose handling after a glucose challenge, whereas ITT evaluates insulin responsiveness. As a result, these measures do not necessarily change in parallel. To better understand the altered fasting phenotype, future studies measuring hepatic glycogen content, gluconeogenic gene expression, and glucagon signaling will help define the underlying metabolic pathways modulating this response in *Gpr37 + /-* mice.

The findings of this study demonstrate GPR37-dependent, sex-biased metabolic effects, with male *Gpr37 + /-* mice exhibiting more pronounced phenotypic changes; however, the exact mechanisms underlying this sex bias remain to be determined. Testosterone, the primary male sex hormone produced by the testes, has been associated with reduced body fat, improved insulin sensitivity, and lower blood glucose levels in men (11) [11]. It also plays a protective role against metabolic syndrome by enhancing cellular responsiveness to insulin (12) [12]. While the insulin tolerance results support improved insulin sensitivity in male *Gpr37 + /-* mice at the physiological level, defining the underlying molecular and tissue-specific mechanisms represents an important next step. The delayed return toward baseline glucose following insulin administration in *Gpr37 + /-* mice likely reflects differences in counter-regulatory responses, such as glucagon, stress hormone signaling, and hepatic glucose production, rather than reduced insulin sensitivity itself. Furthermore, reduced body weight is also closely linked to insulin responsiveness, and the enhanced insulin sensitivity observed in male *Gpr37 + /-* mice may be partly secondary to lower body mass. Future studies incorporating body composition measurements and weight-controlled designs will be needed to distinguish direct GPR37-dependent effects from weight-associated effects. Complementary mechanistic studies using direct measures of insulin signaling, endocrine regulation, and tissue-specific glucose metabolism will help clarify the biological pathways driving this effect.

GPR37 is expressed in the testes, particularly within Sertoli cells, which play a critical role in supporting spermatogenesis and regulating testosterone production. Loss of GPR37 has been shown to impair Sertoli cell proliferation and maturation [13]. Although GPR37 is not expressed in Leydig cells, the primary producers of testicular testosterone, functional crosstalk between Sertoli and Leydig cells is essential for regulating testosterone synthesis. Signals originating from Sertoli cells can either promote or inhibit testosterone production, highlighting their critical role in maintaining androgen homeostasis [14–16]. These observations provide a biologically plausible framework for the sex-biased metabolic phenotypes observed in male *Gpr37 + /-* mice, but this proposed mechanism remains speculative and was not directly tested here. Direct experimental studies will be needed to determine whether GPR37-dependent gonadal or endocrine mechanisms contribute to these effects.

Given the reduced body weight observed in *Gpr37 + /-* male mice, it is also important to consider whether these mice exhibit altered nutrient absorption or gastrointestinal function. GPR37 has been reported to be expressed in multiple regions of the mouse digestive system, including the pancreatic islets of Langerhans, in the nerve plexuses of the esophagus, small intestine, and large intestine [17]. In addition, GPR37 is expressed in enteric glial cells lining the gut wall [18]. Together, these findings suggest that GPR37 could play a role in modulating nutrient uptake and gut homeostasis. Future studies aimed at elucidating the role of GPR37 in the gastrointestinal tract could provide valuable insights into its contribution to metabolic regulation, body weight control, and fasting responses.

This study highlights the potential role for GPR37 in the regulation of metabolism and energy homeostasis, as reflected by sex-biased differences in body weight, fasting responses, and changes in insulin sensitivity. The experiments were conducted in *Gpr37 + /-* mice, a genotype expected to produce reduced receptor dosage, although tissue-specific GPR37 expression levels were not directly measured. The heterozygous model was used here to test whether partial reduction of GPR37 is sufficient to alter metabolic phenotypes, whereas comprehensive metabolic analysis of full knockout mice will be an important next step to define gene-dosage and mechanism-dependent effects. To further elucidate the mechanistic basis of these findings, future studies utilizing full knock-out (*Gpr37 -/-*) mice are warranted. Such models would provide a more complete understanding of the physiological consequences of total GPR37 loss and its impact on metabolic regulation.

Hormonal profiling (including measurements of fasting insulin, glucagon, and testosterone), together with tissue-specific analyses of insulin signaling in liver and muscle will be important next steps to distinguish endocrine, hepatic, and peripheral contributors to the observed phenotypes. Complementary assessment of food intake, energy expenditure, and indirect calorimetry will further clarify whether the body weight and metabolic phenotypes reflect altered energy balance or substrate utilization. Ultimately, these insights could inform the development of novel therapeutic strategies targeting GPR37 for the treatment of metabolic disorders. Pharmacological and tissue-specific genetic studies will be important next steps to test how reduced GPR37 activity affects metabolism across biological contexts.

## Supporting information

S1 File Raw Data. (XLSX)
